# The Treatment of Warts by Internal Administration of Arsenic

**Published:** 1887-12

**Authors:** Bingley G. Pullin

**Affiliations:** Sidmouth


### THE TREATMENT OF WARTS BY INTERNAL ADMINISTRATION OF ARSENIC. By BINGLEY G. PULLIN, M.R.C.S., &c., Sidmouth.

During the last two years many cases of warts on the hands of young children have come under my notice, for treatment; and the following cases will, I think, fully show the efficacy and supreme advantage of arsenic, given internally, over the local application of the powerful caustics :—

C. P., æt 17, a young lady, consulted me in consequence of having suddenly developed innumerable warts on her hands, and which she said had grown with great rapidity, some of the largest of which had attained their growth within a week or ten days ; the whole skin of the hands seemed to be filled with small growths, some not visible, but easily distinguishable to touch. To attack all with nitric acid would have been utterly impossible. I therefore applied the acid to about half a dozen of the largest, and put her on a mixture containing liq. arsenicalis, ♏ iij. twice a day: at the end of a week she came to me in a state of great delight, for after having taken but two bottles of the mixture not a vestige of a wart was to be seen or felt, except the scars left by the application of the acid to the few I treated in this way.

John F., æt 8, was brought to me in consequence of his having several large warts on his hands, and which repeatedly bled. I decided at once to treat him by arsenic internally, and not apply anything externally. He took liq. arsenicalis, ♏ ij. twice a day: at the end of a week all the warts assumed a dull leaden colour. I repeated the medicine, and ordered him to see me again in a week's time; at the end of which, on seeing him, all the warts had disappeared but one, which I easily removed with my finger.

Minnie P., æt 4, I happened to see while attending her mother in a confinement, and was struck by the peculiar appearance of one of her little fingers, the ungual phalanx of which was apparently the seat of some abnormal growth, being irregular and bulbous; on closer examination, however, it proved to be nothing more than a crop of warts, which almost entirely covered the nail. On the same hand there were many others, and likewise on the opposite hand. After taking liq. arsenicalis, ♏ j. for ten days, most of the warts had fallen off, and those which still remained I easily removed with my finger and without the slightest pain to the child. Several other cases, in which I have treated these unsightly and troublesome growths in the same way, I could record, but in every instance the cure has been complete and rapid; and I think the above cases in themselves alone prove the value of arsenic for treating warts; for it is certainly equally rapid in action, and moreover has the great advantage over the caustics in being painless, which is of so much importance in treating young children.

